# Supplementary material for: The causal relationship between the human gut microbiota and pyogenic arthritis: a Mendelian randomization study
Source: Front Cell Infect Microbiol. 2024 Nov 26;14:1452480. doi: 10.3389/fcimb.2024.1452480 (PMC11629706; doi:10.3389/fcimb.2024.1452480)
Supplement: Supplementary file 1 [file DataSheet1.zip › mendelian test/ebi-a-GCST90027602.csv_leave_one_out.pdf]

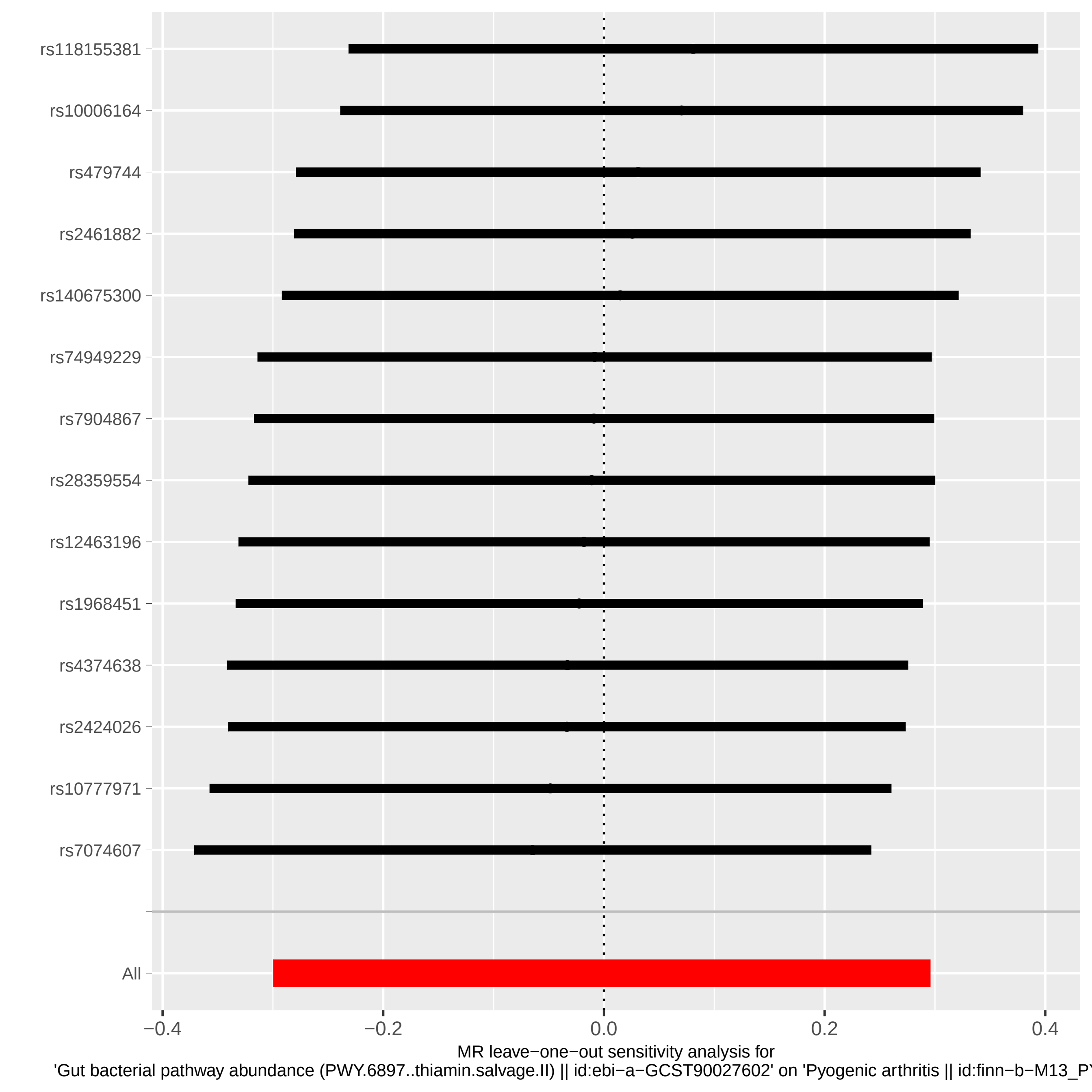
rs118155381
rs10006164
rs479744
rs2461882
rs140675300
rs74949229
rs7904867
rs28359554
rs12463196
rs1968451
rs4374638
rs2424026
rs10777971
rs7074607
All
−0.4
−0.2
0.0
0.2
0.4
MR leave−one−out sensitivity analysis for
'Gut bacterial pathway abundance (PWY.6897..thiamin.salvage.II) || id:ebi−a−GCST90027602' on 'Pyogenic arthritis || id:finn−b−M13_P
